# Endoperoxide‐enhanced self‐assembled ROS producer as intracellular prodrugs for tumor chemotherapy and chemodynamic therapy

**DOI:** 10.1002/EXP.20230127

**Published:** 2024-02-09

**Authors:** JunJie Tang, Yadong Liu, Yifan Xue, Zhaozhong Jiang, Baizhu Chen, Jie Liu

**Affiliations:** ^1^ School of Biomedical Engineering Shenzhen Campus of Sun Yat‐Sen University Shenzhen Guangdong People's Republic of China; ^2^ Department of Biomedical Engineering Integrated Science and Technology Center Yale University West Haven Connecticut USA; ^3^ Guangdong Provincial Key Laboratory of Sensor Technology and Biomedical Instrument Sun Yat‐Sen University Guangzhou China

**Keywords:** artesunate, chemodynamic therapy, prodrug‐based self‐assembled nanoparticles, reactive oxygen species producer

## Abstract

Prodrug‐based self‐assembled nanoparticles (PSNs) with tailored responses to tumor microenvironments show a significant promise for chemodynamic therapy (CDT) by generating highly toxic reactive oxygen species (ROS). However, the insufficient level of intracellular ROS and the limited drug accumulation remain major challenges for further clinical transformation. In this study, the PSNs for the delivery of artesunate (ARS) are demonstrated by designing the pH‐responsive ARS‐4‐hydroxybenzoyl hydrazide (HBZ)‐5‐amino levulinic acid (ALA) nanoparticles (AHA NPs) with self‐supplied ROS for excellent chemotherapy and CDT. The PSNs greatly improved the loading capacity of artesunate and the ROS generation from endoperoxide bridge using the electron withdrawing group attached directly to C10 site of artesunate. The ALA and ARS‐HBZ could be released from AHA NPs under the cleavage of hydrazone bonds triggered by the acidic surroundings. Besides, the ALA increased the intracellular level of heme in mitochondria, further promoting the ROS generation and lipid peroxidation with ARS‐HBZ for excellent anti‐tumor effects. Our study improved the chemotherapy of ARS through the chemical modification, pointing out the potential applications in the clinical fields.

## Graphical abstract

To overcome the pharmacological limitations of artemisinin in cancer therapy, the novel pH‐responsive artesunate (ARS)‐4‐hydroxybenzoyl hydrazide (HBZ)‐5‐amino levulinic acid (ALA) nanoparticles (AHA NPs) with self‐supplied capacity of reactive oxygen species (ROS) were reported to realize excellent chemotherapy and chemodynamic therapy (CDT).

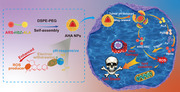

## 1 INTRODUCTION

Chemodynamic therapy (CDT) is a revolutionary antitumor therapeutic method using Fenton reaction with peroxide groups for the high level of toxic reactive oxygen species (ROS) to induce cell apoptosis.^[^ ^1^, ^2^, ^3^ ^]^ CDT does not require oxygen (O_2_) or the external light source, and thus overcome the drawbacks of photodynamic treatment (PDT) as well as sonodynamic therapy (SDT), such as hypoxia resistance or the limited laser penetration.^[^ ^4^, ^5^, ^6^ ^]^ The CDT technique is still in its infancy despite its well tumor selectivity and few side effects.^[^ ^7^, ^8^ ^]^ Currently, the common method used in CDT is to administrate low‐valent transition metal ions with high Fenton reaction activity for the generation of hydrogen peroxide (H_2_O_2_) intracellularly and further transformation into highly reactive hydroxyl radicals, leading to high oxidative stress and subsequently cell death.^[^ ^9^, ^10^, ^11^, ^12^ ^]^ However, a major issue of this technique is that exogenous injection of the excessive Fenton‐type heavy metals, such as iron, manganese, copper, or cobalt, may have side effects, including acute or chronic poisoning.^[^ ^11^, ^13^, ^14^, ^15^ ^]^ Therefore, it is still highly desirable to find an alternative approach to overcome this problem in CDT.

The prodrug‐based self‐assembled nanoparticle (PSN) is a novel self‐delivery nanoplatform formed by preprocessing drug molecules into prodrugs and then self‐assembly into nanoparticles.^[^ ^16^, ^17^, ^18^ ^]^ The PSNs have several benefits over conventional nanocarriers (liposomes, micelles, albumin, and polymers), such as a larger capacity for drug loading, improved biosafety, and less physiological and psychological stress.^[^ ^19^, ^20^, ^21^ ^]^ The prodrugs act as both transporters and cargo with the high loading capacity for chemotherapeutics. The chemotherapeutic drugs in homo‐ or hetero‐dimer strategy with unique sensitivity linkages have been demonstrated to form PSNs, which attracted significant scientific interests in recent years.^[^ ^22^ ^]^ PSNs are able to target and kill the tumor cells precisely by reacting to the greatly increased ROS/GSH (glutathione) levels and acidic endoplasmic pH in TME with seldom side effects on normal organs.^[^ ^23^ ^]^

For decades, the preparation of artemisinin, a natural component from the Chinese traditional herb *Artemisia annua* L. with an endoperoxide bridge, has served as a crucial frontline medication against superficial malarial infections.^[^ ^24^, ^25^ ^]^ Besides the well‐known antimalarial properties, artemisinin has also shown desirable cytotoxicity against a range of cancer types with a significantly higher intracellular iron pool, since free radicals generated from iron‐mediated breakage of the endoperoxide bridge allows a selective kill of cancer cells.^[^ ^26^, ^27^, ^28^, ^29^, ^30^ ^]^ However, the clinical applications of artemisinin still suffer from the short half‐life, poor solubility, low bioavailability, and the moderate activity against cancer cells.^[^ ^31^, ^32^, ^33^ ^]^ To overcome the pharmacological limitations of artemisinin, numerous artemisinin analogues for enhancing ROS generation have been explored by the electron withdrawing group attached directly to C10 of artemisinin.^[^ ^34^, ^35^, ^36^, ^37^ ^]^ For instance, Zhang et al. designed and synthesized a derivative, ART‐TPP, which was also capable to target the mitochondria and exerted more potent inhibition than artesunate to cancer cells.^[^ ^38^ ^]^ In order to compensate for the insufficient therapeutic effect of artemisinin, Wang et al. combined the gemcitabine with artemisinin through thioacetal bond to achieve ROS responsive drug release in vivo, combining chemotherapy and CDT.^[^ ^39^ ^]^ Recent studies indicated that heme, rather than the free ferrous iron, predominately contributed to the activation of artemisinin, producing reactive free radicals that could alkylate a variety of cellular targets promiscuously.^[^ ^38^, ^40^, ^41^, ^42^, ^43^ ^]^ In tumor cells, various porphyrin oxidase enzymes subsequently catalyze the ALA to protoporphyrin (PpIX) for further combination with intracellular Fe^2+^ to generate heme.^[^ ^44^, ^45^, ^46^, ^47^ ^]^ Since the concentration of intracellular iron in cancer cells has already been at a high level, the addition of ALA rather than free ferrous alone plays a key role in the formation of heme.^[^ ^48^, ^49^, ^50^ ^]^

**SCHEME 1:**
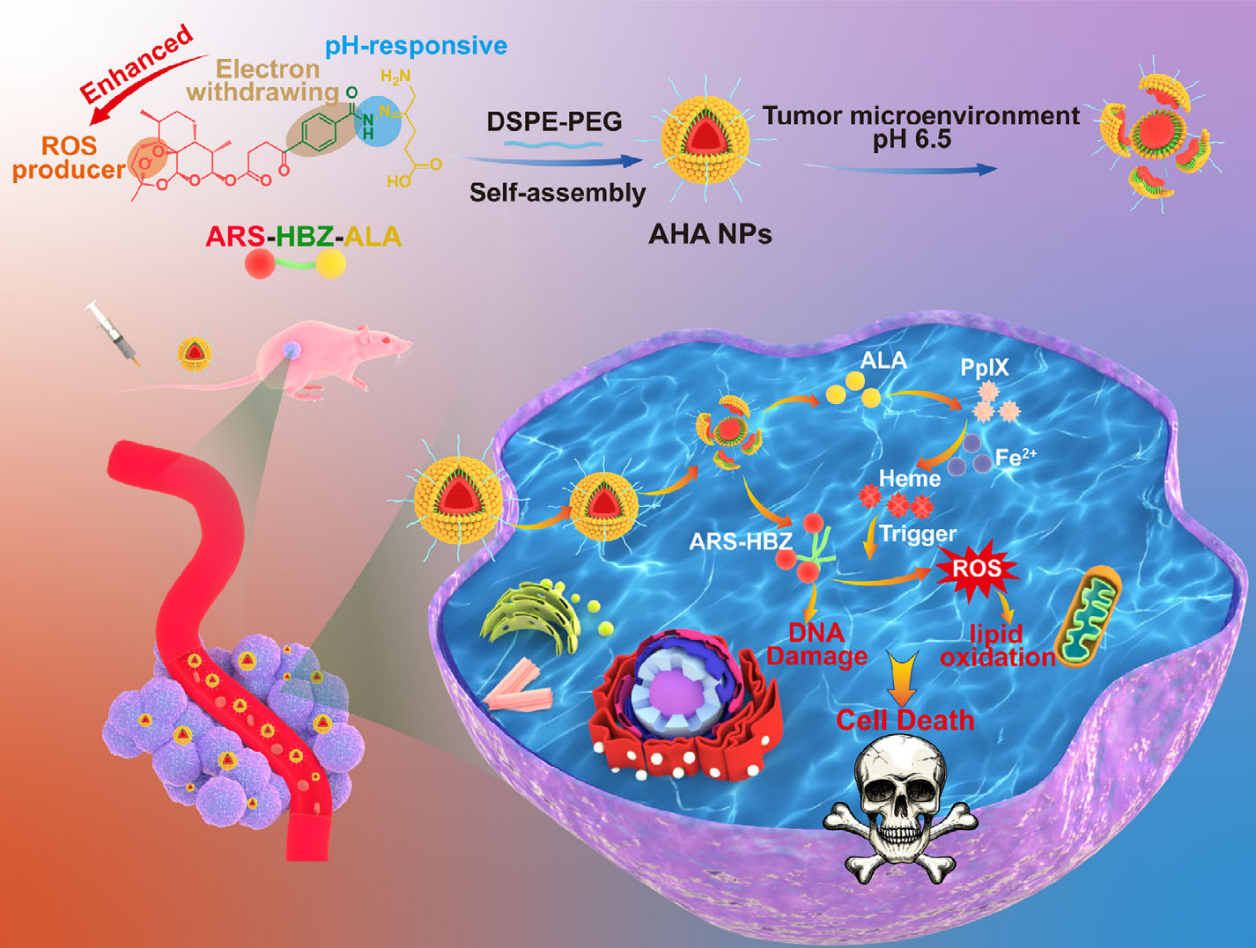
The endoperoxide‐enhanced self‐assembled reactive oxygen species (ROS) producer as intracellular prodrugs for tumor chemotherapy andchemodynamic therapy (CDT).

To overcome the pharmacological limitations of artemisinin, in this study, we reported a novel pH‐responsive artesunate (ARS)−4‐hydroxybenzoyl hydrazide (HBZ)−5‐amino levulinic acid (ALA) nanoparticles (AHA NPs) with self‐supplied capacity of ROS to realize excellent chemotherapy and CDT (Scheme 1). The PSNs greatly improved the loading capacity of artesunate and the ROS generation from endoperoxide bridge using the electron withdrawing group attached directly to C10 of artesunate. As the PSNs system, AHA NPs presented the long blood circulation and well tumor target ability due to the enhanced permeability and retention (EPR) effect. The pH responsive AHA NPs could release ARS‐HBZ and ALA. The nanosized artemisinin derivatives showed better antitumor effect than free drug in liver cancer tumor‐bearing mouse model. When reaching the tumor sites, ALA and ARS‐HBZ could release quickly after internalized by tumor cells owing to the dissociation of hydrazone bonds triggered by the drop of pH value in lysosomes, while presented limited side effects on normal tissues. Compared to artesunate, ARS‐HBZ showed better ROS production capacity under the electron withdrawing effect of HBZ. Additionally, ALA boosted the amount of heme inside cells through a series of enzymatic catalytic events in the mitochondria, leading to the promotion of ROS lipid peroxidation‐related membrane damage and cell death when combining with ARS‐HBZ. The synergy of ARS‐HBZ and ALA achieved better anti‐tumor effects as the PSNs system. The in vitro cellular uptake rate, ROS generation and cytotoxicity of AHA NPs tests showed the excellent antitumor efficacy. The biodistribution, antitumor effect, and biosafety of the NPs were also examined in vivo to evaluate their potential application in CDT treatment. The AHA NPs were evaluated for their antitumor effect in vitro and in vivo. Our study performed the multifunctional and artemisinin derivative AHA NPs, pointing out a novel chemical modification on artemisinin prodrugs for further clinical transformation prospects.

## 2 EXPERIMENTAL SECTION

### 2.1 Materials, cell culture and animals

Artemisinin and its derivates (artemether, dihydroartemisinin and artesunate) were purchased from Aladdin Chemistry Co., Ltd. 5‐amino levulinic acid (ALA, purified 99%), 4‐hydroxybenzhydrazide (HBZ, purified 99%), heme and di‐tert butyl dicarbonate ((Boc)_2_O) were purchased from Sigma‐Aldrich (St. Louis, MO, USA). Methanol, dichloromethane (DCM), *N*,*N*‐dimethylformamide (DMF), trifluoroacetic acid (TFA), 4‐dimethylaminopyridine (DMAP) and dicyclohexylcarbodiimide (DCC) were purchased from Aldrich Chemical Co. 1,2‐distearoyl‐sn‐glycero‐3‐phosphoethanolamine‐polyethylene glycol‐2000 (DSPE‐PEG2000) was obtained from AVT (shanghai) Pharmaceutical Tech Co., Ltd. Thiazolyl Blue Tetrazolium Bromide (MTT), 4′,6‐diamidino‐2‐phenylindole (DAPI), reactive oxygen species assay kit (DCFH‐DA) and Annexin V‐FITC/PI Apoptosis Detection Kit were purchased from Shanghai BiYuntian Biotechnology Co., Ltd.

Dulbecco's Modified Eagle Medium (DMEM, Hyclone) was used to cultivate HepG2 tumor cell lines purchased from the Shanghai Cell Bank of the Chinese Academy of Sciences (Shanghai, China). BALB/C male nude mice (2–4 weeks, 15 g) were acquired and reared at Sun Yat‐sen University's Center for Experimental Animals. The Animal Care and Use Committee of Sun Yat‐sen University approved all animal research.

### 2.2 Instruments

The Bruker AVANCE III HD 400 MHz was used to measure the NMR spectra. The Nano Zetasizer (Malvern, Nano ZS90, Britain) was used to test the particle size and potential. The transmission electron microscope (TEM, H7650, Japan) was used to observe the morphologies. A spectrophotometer (DU‐730, USA) was used to get the UV spectrum. The microplate reader (BioTek Synergy4, USA) was used to assess the cell viability. The confocal laser scanning microscope (CLSM, FV3000, Olympus) was used to capture the cell fluorescence pictures. The cell fluorescence quantitative test was carried out by flow cytometry using a BD FACSCalibur device. An optical microscope (Motic AE31, Xiamen) was used to detect the tissue slices.

### 2.3 Synthesis and characterization of AHA NPs

#### 2.3.1 Synthesis of ARS‐HBZ‐ALA

**SCHEME 2:**
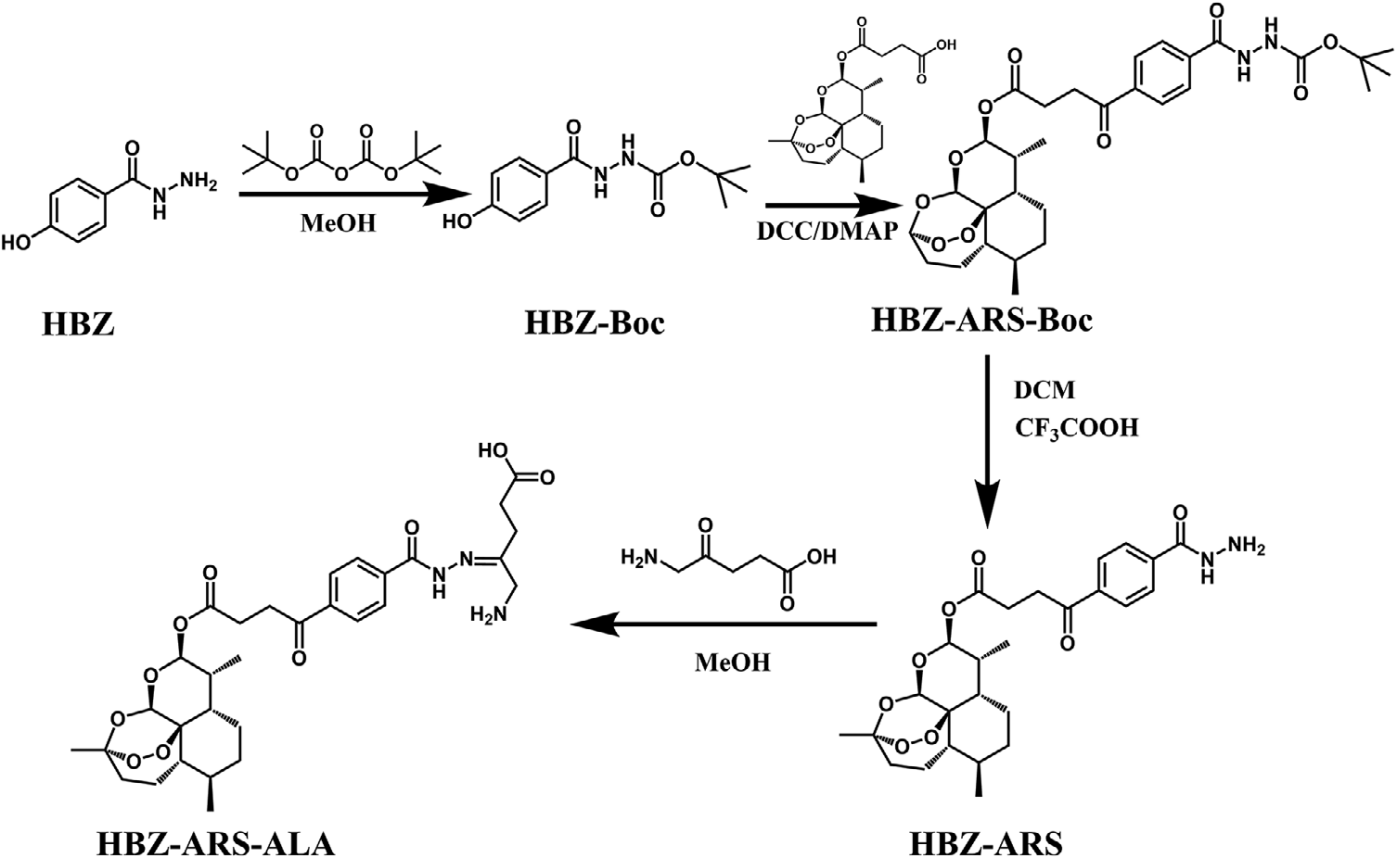
Synthesis of artesunate (ARS)−4‐hydroxybenzoyl hydrazide (HBZ)−5‐amino levulinic acid (ALA).

ARS‐HBZ‐ALA was synthesized according to Scheme 2. First, 10 mL of methanol was used to dissolve HBZ (1.6 mmol, 243.4 mg) and (Boc)_2_O (1.6 mmol, 349.2 mg) before the mixture was agitated at room temperature for 24 h. The suspension was recrystallization and the resulting white solid (HBZ‐Boc) was washed with DCM.

In a flask containing 10 mL of DMF, ARS (1.6 mmol, 615.1 mg), DCC (1.6 mmol, 330.1 mg) and DMAP (0.4 mmol, 44.9 mg) were added sequentially. The solution was then agitated at 0°C for 30 min. The mixture was subsequently supplemented with HBZ‐Boc (1.6 mmol, 403.6 mg), and stirred at room temperature overnight. The crude product was eluted with DCM/MeOH (20:1) after the rotational evaporation of the solvent, resulting in ARS‐HBZ‐Boc as a white solid (764.1 mg, 70.1%).

Subsequently, ARS‐HBZ‐Boc (1.6 mmol, 829.9 mg) was dissolved in DCM/TFA (1:1) and the mixture was agitated at room temperature for 2 h. The crude product was eluted with DCM/MeOH (10:1) after the rotational evaporation of the DCM/TFA solvent was eliminated using rotary evaporation, yielding ARS‐HBZ as a white solid (543.6 mg, 65.5%).

Then, ARS‐HBZ (1.6 mmol, 858.5 mg) and ALA (2.4 mmol, 314.7 mg) were dissolved in 10 mL of DCM and the solution was stirred at room temperature for 24 h. After the rotational evaporation of the solvent, the crude product was further purified by eluting with DCM/MeOH (20:1) to obtain ARS‐HBZ‐ALA as a buff solid (727.4 mg, 62%). Finally, the ARS‐HBZ‐ALA was collected and further confirmed by ^1^H‐NMR (Figure S1).

#### 2.3.2 Synthesis of ARS‐HBZ‐ALA (AHA) NPs

The self‐assembled approach was used to form the AHA NPs. ARS‐HBZ‐ALA (2 mg) and DSPE‐PEG2000 (1 mg) were dissolved in 500 µL of THF, vigorously stirred and then slowly added to 2 mL of PBS (pH = 7.4). To avoid the influence of THF, the resultant mixture was agitated for an additional 12 h at room temperature. All samples were directly analyzed and measured at 25°C. DLS (Malvern Zetasizer Nano ZS) and TEM (Hitachi H‐7650) were used to study the size distribution and morphology of the AHA NPs.

#### 2.3.3 The pH responsive property of the AHA NPs

The temperature was kept at 37°C. Different pH (7.4, 6.5) conditions were used to investigate the size change of the pH‐responsive AHA NPs. In a nutshell, AHA NPs were dialyzed against PBS in various pH level at 37°C. Using DLS and TEM, the morphology and particle size distribution of the NPs were observed.

The pH‐activated drug release performance of the AHA NPs was investigated using HPLC (Agilent, C18 reverse phase column, MeOH and double distilled water, v:v = 80:20). Briefly stated, AHA NPs were dissolved in 5 mL of water and put in a dialysate bag (MwCO = 3500). The release media was added to the dialysis bag, and then submerged with constantly stirring at a speed of 100 rpm. The fresh medium in the same volume was introduced at regular intervals after collecting samples (2 mL) from the outside of the dialysis bag. Lastly, HPLC was used to assess the content of ART in the released media. The concentration of each sample was evaluated in triplicates.

#### 2.3.4 AHA NPs interaction assays

Each experiment was carried out at room temperature. Ammonium acetate or phosphate buffer was used to perform absorption spectra in a 1‐mL cuvette. In a nutshell, a mixture of 10 µL of heme (2 mm), 10 µL of AHA NPs solution at different concentrations and 980 µL of PBS (0.2 m, pH = 5.0) was used to analyze the spectrum.

The heme‐competition experiments were conducted in microplates containing 20 µm of heme, 40 µm of AHA NPs, and various concentrations of ferrocyanide or ferrous chloride at a range of inorganic iron to heme molar ratios (0, 5, 10, 20, 40 and 80) in PBS (pH = 5.0) buffer with 30% of DMSO (v/v). The A_416_ of the sample without heme was used as no inhibition (100% of reduction) of AHA NPs activation, and the absorbance of the sample without AHA NPs (but with heme and ferrocyanide) was taken as complete inhibition (0% of reduction) in activation.

#### 2.3.5 ROS generation in solution

The efficiency of ROS production in AHA NPs was calculated using DPBF as the probe. In a nutshell, 10 µL of heme (2 mm) and 10 µL of AHA NPs solution were combined with 980 µL of PBS (pH = 6.5) solution containing 10 mm DPBF. The working concentration of ARS and ARS‐HBZ was 20 µg mL^−1^. The working concentration of AHA NPs was 30 µg mL^−1^, and the absorbance of DPBF was measured at 413 nm by a UV detector.

#### 2.3.6 Theoretical calculation

The geometries and electrical structures were revealed by the density functional theory (Gaussian B3LYP) computations. The energy levels and border molecular orbitals were calculated.

### 2.4 Cellular experiments

#### 2.4.1 Cellular uptake

ALA is an inborn precursor of the red‐fluorescent photosensitizer PpIX. Therefore, we investigated the uptake of AHA NPs in HepG2 cells by detecting the production of intracellular PpIX. For an overnight incubation, HepG2 cells (3 × 10^5^ cells per mL) were placed into a 6‐well culture plate. The adherent cells were treated with fresh DMEM media containing AHA NPs or free ALA (ALA concentration = 40 µm) for a series of time points (1 h, 2 h, 4 h, 6 h, 8 h and 12 h) after achieving a confluence of 70%. The HepG2 cells were then obtained by trypsinization, centrifuged and PBS‐washed. Using flow cytometry (Becton Dickinson, San Jose), PpIX fluorescence in single‐cell suspension was evaluated. CLSM (Olympus) was used to study the AHA NPs' intracellular distribution. In a nutshell, HepG2 cells (3 × 10^5^ cells per mL) were seeded in the confocal bottom plates and treated for 6 h with either free ALA (40 µm) or AHA NPs. The HepG2 cells were then stained with DAPI and visualized using CLSM.

#### 2.4.2 Cellular ROS generation

HepG2 cells were plated in the confocal bottom dishes with a density of 3 × 10^5^ cells per mL for 24 h in order to detect the ROS generation intracellularly. Six groups were created by randomly dividing the adhering cells: control, ARS, ARS‐HBZ, ARS+ALA, ARS‐HBZ+ALA, and AHA NPs (40 µm). The HepG2 cell groups were cultivated under their respective treatment conditions for 6 h before the culture medium was changed to 20 µm of DCFH‐DA to react with intracellular ROS for 20 min. The DCF fluorescence intensity of all groups in single‐cell suspension was objectively quantified and visualized under CLSM.

#### 2.4.3 Intracellular iron/heme concentration test

In a 6‐well plat, HepG2 cells was cultured for 24 h at 37°C in a humidified environment with 5% of CO_2_. Cells were split into five groups (Control, ARS, ARS‐HBZ, ALA, and AHA NPs) and incubated overnight. The cells from different groups were incubated for 24 h, then washed three times by ice‐cold PBS before collected in PBS solution. For cell lysis by ultrasonication (25% amplification), equal numbers of cells in different groups were employed. After centrifugation (14,000 × *g*, 10 min, 4°C), the absolute value of iron was evaluated using a cell iron content assay kit (Solarbio), following the manufacturer's instructions. A commercial heme assay kit (Abnova) was used to determine absolute amount of heme in each sample according to the manufacturer's instructions.

#### 2.4.4 Cytotoxicity

The in vitro cytotoxicity of AHA NPs on HepG2 cells was detected using the standard MTT method. In a 96‐well plate, HepG2 cells (5 × 10^3^ cells per well) were seeded and incubated for 24 h at 37°C in a humidified environment with 5% of CO_2_. The cells in each well were treated by different samples with ART concentrations (5, 10, 20, 40 and 80 µm) and then cultured overnight. The culture medium was changed to the 120 µL of fresh media containing 20 µL of MTT (5 mg mL^−1^) solution and cultured for another 4 h. The crystallized formazan salt was then dissolved in DMSO and measured using a microplate reader (BioTek Synergy 4). The relative cell viability was determined using the following formula:

$$\mathrm{Cell}\;\mathrm{viability}\left(\%\right)=\frac{A_{\mathrm{sample}}-A_{\mathrm{blank}}}{A_{\mathrm{control}}-A_{\mathrm{blank}}}\times 100\%$$

where $A_{\mathrm{blank}}$ was the absorbance at 570 nm without any cells. $A_{\mathrm{control}}$ and $A_{\mathrm{sample}}$ were the absorbance values obtained from the samples with and without NPs, respectively.

#### 2.4.5 Cell apoptosis assay

HepG2 cells (3 × 10^5^ cells per mL) were plated in the 6‐well plates for adherence overnight to study the apoptosis caused by the AHA NPs. The ARS, ARS‐HBZ, ALA, and AHA NPs were treated with the adhering cells for 6 h. After incubation for 24 h, the HepG2 cells were collected by trypsinization, centrifuged, and washed by PBS. The cells were then collected, labeled for 15 min in the dark with 5 µL of AnnexinV‐FITC and propidium iodide (PI), and examined by flow cytometry.

#### 2.4.6 Cell cycle assay

The cell cycle analysis procedure was identical to the apoptosis test described above. HepG2 cells (3 × 10^5^ cells per mL) were seeded and handled similarly for the cell apoptosis investigation. After incubation for 30 min in the dark, the HepG2 cells were resuspended in PBS, labeled with PI, and subjected for flow cytometry analysis.

### 2.5 In vivo antitumor experiments

#### 2.5.1 Biodistribution

10^7^ of HepG2 cells were subcutaneously injected into the lateral abdomen of the mice to create the subcutaneous tumor model. The studies were conducted after the tumor volume reaching 100 mm^3^. Cy5.5 was utilized as a fluorescent marker to examine the distribution of AHA NPs in vivo using a small animal imaging system (IVIS Lumina XR Series III, PerkinElmer). Intravenously administered free Cy5.5 and AHA@Cy5.5 injections at a Cy5.5 dosage of 0.4 mg kg^−1^ were given to the tumor‐bearing nude mice. The imaging device captured the fluorescence signals of the mice at different time points (4, 8, 12, 24, 48 and 72 h) after injection. After 24 h, the tumors and healthy organs were removed during dissection. Using the ex vivo imaging technique, the fluorescence intensity of organs was identified and measured.

#### 2.5.2 In vivo antitumor activity

The tumor‐bearing mice, after tumor volume reaching ≈100 mm^3^, were randomly assigned into six groups (*n* = 5) to examine the anticancer activity in vivo as follows: (i) 0.9% of NaCl solution (Control), (ii) free ARS, (iii) free ARS‐HBZ, (iv) free ALA, and (v) AHA NPs. On Day 0, 3, and 6, the mice received intravenously administered doses of various medication formulations containing ARS (20 mg kg^−1^), ALA (8 mg kg^−1^) and AHA NPs (30 mg kg^−1^). The body weight and tumor volume changes were noted every two days over the course of the medication. The percentage of mice which died or bore the tumor with volume larger than 1500 mm^3^ was used to create the Kaplan–Meier survival curves. Tumors and major organs from the mice were removed, weighed and photographed on the 22nd day. To assess the tumor suppressive effect of the AHA NPs, the results of hematoxylin and eosin (H&E) and TUNEL staining were analyzed.

#### 2.5.3 Biosafety

After the cancer therapy, the major organs of treated mice were collected and stained by H&E to assess the potential adverse effects of the AHA NPs. The concentrations of alanine aminotransferase (ALT), alkaline phosphatase (ALP), aspartate transaminase (AST), urea nitrogen (BUN), creatinine (CR), and creatine kinase (CK) were measured in blood samples taken through the ocular puncture.

### 2.6 Statistical analysis

To determine the means and the standard deviation, each experiment was at least in triplicates. The experimental data were examined using the *t*‐test, and ^*^ *p* < 0.05, ^**^ *p* < 0.01 and ^***^ *p* < 0.001 declared the statistically significant.

## 3 RESULTS AND DISCUSSIONS

### 3.1 Synthesis and characterization of AHA NPs

**FIGURE 1:**
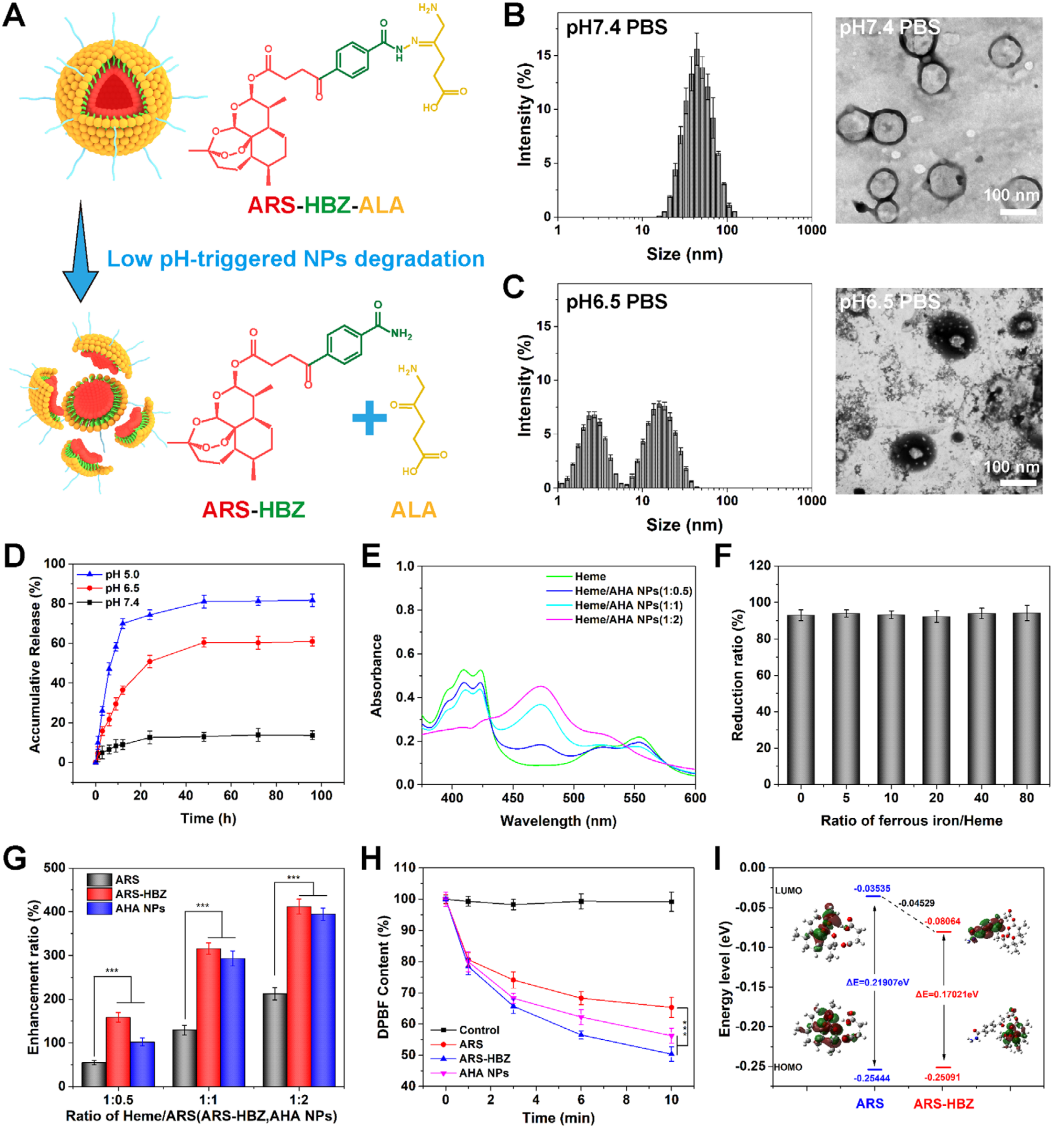
Synthesis and characterization of artesunate (ARS)−4‐hydroxybenzoyl hydrazide (HBZ)−5‐amino levulinic acid (ALA) nanoparticles (AHA NPs). (A) The pH‐response of AHA NPs. Size distribution and transmission electron microscope (TEM) images of AHA NPs incubated with (B) PBS (pH = 7.4) and (C) PBS (pH = 6.5). (D) Drug release profiles of AHA NPs in vitro. (E) Activation of AHA NPs by heme. (F) Competition of ferrous iron with heme in the activation of AHA NPs. (G) Activation of ARS, ARS‐HBZ and AHA NPs by heme. (H) Reactive oxygen species (ROS) generation by heme activation of ARS, ARS‐HBZ and AHA NPs. (I) Calculated HOMO/LUMO energy and energy gap (Δ*E*) of ARS and ARS‐HBZ molecule. Data are given as mean ± S.D. (*n* = 3). *p < 0.05, **p < 0.01 and ***p < 0.001. Scale bar = 100 nm.

The synthesis of ARS‐HBZ‐ALA was shown in Scheme 2. Firstly, (Boc)_2_O was used to protect the amino group at one end of 4‐hydroxybenzoyl hydrazine (HBZ) to obtain the amino‐protected HBZ‐Boc, which prevented the amino group from interfering with the following reaction. Subsequently, DCC/DMAP was used to catalyze the esterification reaction between artesunate (ARS) and HBZ‐Boc, resulting in ARS‐HBZ‐Boc. Boc was removed in a DCM solution of trifluoroacetic acid to obtain ARS‐HBZ. Finally, 5‐aminolevulinic acid (ALA) was connected to the amino group of ARS‐HBZ through a Schiff base reaction to obtain the prodrug ARS‐HBZ‐ALA. The molecular structure of each intermediate in the synthesis step was analyzed by the ^1^H NMR spectrum (Figure S1). Results of transmission electron microscopy (TEM) and dynamic light scattering (DLS) further demonstrated the diameters and spherical structure of the AHA NPs. The hydrazone bonds in AHA NPs could be fractured under acidic conditions, releasing free ARS‐HBZ and ALA, thus destroying the nanostructure (Figure 1A). The AHA NPs were consistently spherical with ≈50 nm‐sized entities, as seen in Figure 1B. Notably, the AHA NPs were stable for 7 days in PBS containing 10% of FBS (v/v, Figure S2). After co‐incubating the prepared AHA NPs with PBS at the pH value of 6.5 for 24 h, DLS and TEM were used to identify changes in the particle size distribution as well as morphology (Figure 1C). As shown, AHA NPs lost the uniform spherical structure under normal conditions and formed smaller nanoparticles. The original plump structure also shrunk significantly. Under acidic conditions, the hydrazone bond inside AHA NPs presented a responsive break, which caused the damage of the nanoparticles and the release of the ARS‐HBZ together with the ALA. The AHA NPs could be used as responsive PSNs in the tumor microenvironment to kill tumor cells.

The drug delivery system was incubated in PBS at various pH values (7.4, 6.5 and 5.0) to evaluate the procedural controlled release behavior of AHA NPs. Figure 1D demonstrated that the cumulative medication release of AHA NPs at the pH value of 7.4 was rather sluggish. When the pH value further decreased, the rate of drug release increased notably. The rapid dissolution of the NPs for ARS‐HBZ and ALA release was attributed to the break of the hydrazone connection of ALA to AHA NPs for the release. Additionally, the controlled release during the procedure at the tumor site resulted in an efficient combination of ALA and ARS‐HBZ treatment.

The interaction with AHA NPs were observed by the Soret and Q absorption bands of heme. The absorption spectra were studied after adding heme at a final concentration of 20 µm with or without AHA NPs at various molar ratios. Figure 1E showed that the heme's Soret band appeared at 416 nm. Under the function of the AHA‐NPs, heme decreased in a concentration‐dependent manner at 416 nm and formed a new absorption peak at 478 nm in the acidic environment. The absorption signal at 416 nm almost entirely disappeared as the AHA NPs: heme molar ratio reached 2:1.

Next, we evaluated the interaction between the AHA NPs and heme. As seen in Figure 1F, ferrocyanide was unable to impede the interaction between heme and AHA NPs even with an 80‐fold of excess. Results revealed that heme interacted with AHA NPs far more effectively than that of the inorganic iron under the in vitro circumstances. Thus, it was of great importance to convert the inorganic iron in the tumor area into heme to bind with artemisinin derivatives. We investigated the effect of Fe^2+^ to the AHA NPs system in Figure S3. Although individual Fe^2+^ could also promote the ROS production of AHA NPs, the production efficiency was significantly lower than that of heme at the same concentration. In addition, we further tested the heme binding ability of the synthetic artemisinin derivative ARS‐HBZ compared with ARS. After mixing in PBS buffer (0.2 m, pH = 5.0), the absorption of heme (20 µm) at 478 nm were examined with ARS, ARS‐HBZ, and AHA NPs at different molar ratios. According to Figure 1G, the absorbance of heme continuously increased along with a concentration dependent manner caused by ARS, ARS‐HBZ and AHA NPs. Moreover, we also found that ARS‐HBZ exhibited better reactivity with heme than that of ARS.

Since the production of ROS was crucial for AHA NPs, DPBF was employed as a probe to identify the level of ROS promoted by heme in AHA NPs. As shown in Figure 1H, ARS, ARS‐HBZ, and AHA NPs performed desired capacity to generate sufficient ROS, with the absorbance of DPBF reduced steadily. Compared to AHA NPs, free ARS‐HBZ exhibited better ROS production ability, because the hydrazone bond inside AHA NPs could not be completely broken in a short period to release ARS‐HBZ and react with heme. More importantly, ARS‐HBZ presented a better ROS production than that of ARS, which was also consistent with the result in Figure 1G.

In order to explore the reason why ARS‐HBZ showed better heme binding ability and ROS production ability than that of ARS, we calculated the orbits and energy levels of the two molecules. The capacity of the chemical to give or accept electrons was shown by FMOs like HOMO and LUMO. The HOMO and LUMO energies could be used to describe the chemical hardness, reactivity, electronegativity, and optical polarizability of any molecule. Figure 1I displayed the HOMO and LUMO energies determined from B3LYP/6‐31G*. The enormously electron‐withdrawing groups were frequently added to reduce the LUMO energy to enhance the capacity to acquire an electron due to the association between reduction potential and the LUMO energy of organic compounds. Therefore, a higher electron affinity was associated with a lower LUMO energy. By reducing the LUMO energy of ARS‐HBZ (0.08064 eV) in contrast to ARS (0.03535 eV), the addition of HBZ might increase the affinity of electrons for a simple and quick electron‐accepting process. Moreover, the total energy difference between HOMO and LUMO was narrowed, which enhanced the electrical conductivity of the system for a more effective response.

### 3.2 Cellular uptake and ROS generation

**FIGURE 2:**
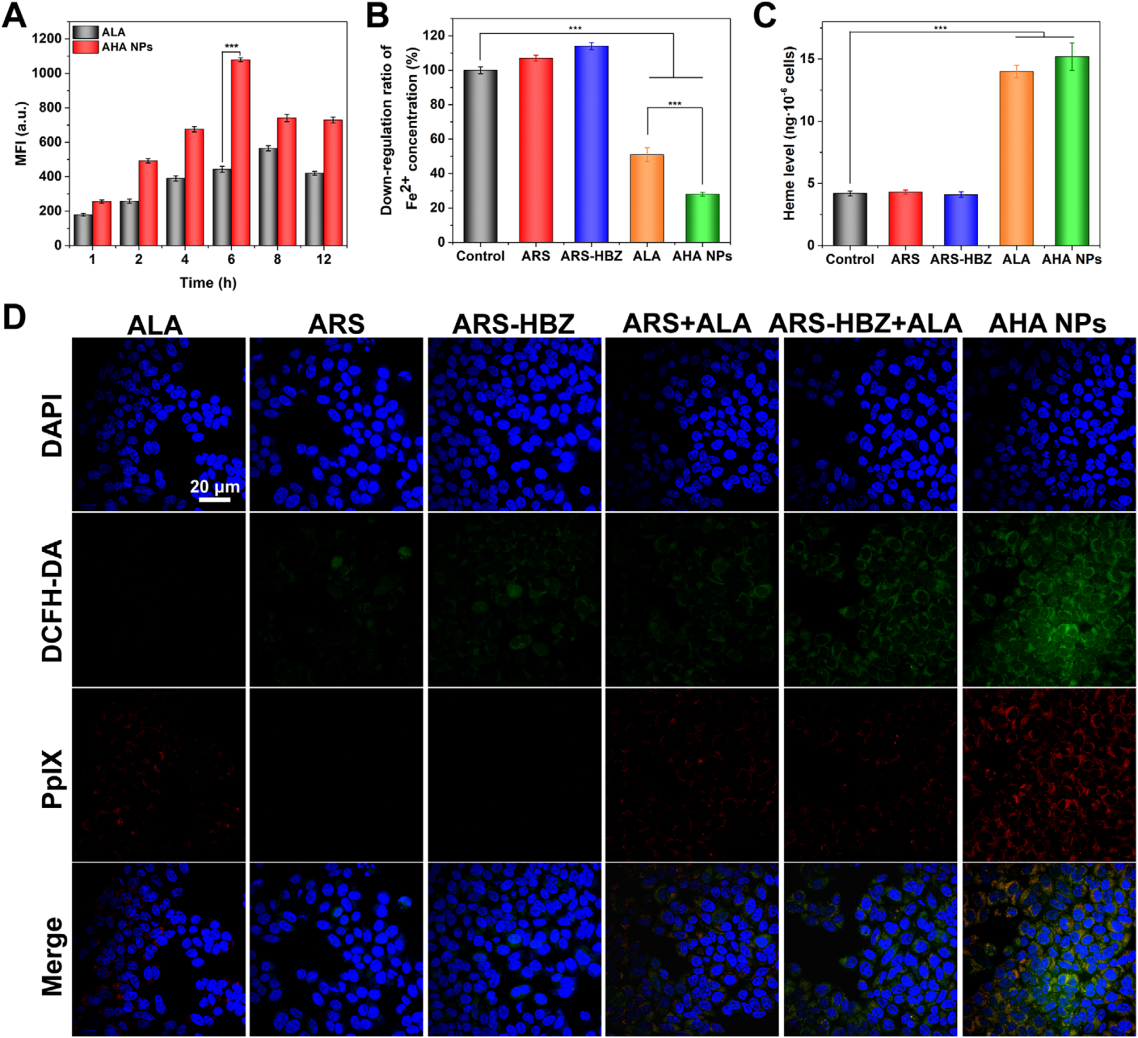
Cellular uptake and ROS generation of AHA NPs. (A) The intracellular mean fluorescence intensity (MFI) of protoporphyrin (PpIX) in HepG2 cells treated with free ALA and AHA NPs. Assessment of (B) intracellular iron and (C) heme in HepG2 cells at 24 h. (D) The confocal laser scanning microscope (CLSM) images of the ROS produced by AHA NPs incubated with of HepG2 cells. Data are given as mean ± S.D. (*n* = 3). *p < 0.05, **p < 0.01 and ***p < 0.001. Scale bar = 20 µm.

ALA was an endogenous precursor of the potential red fluorescence‐producing photosensitizer PpIX. Therefore, the uptake and generation of PpIX from AHA NPs via HepG2 cells were investigated. Figure 2A and Figure S4A illustrated the cellular uptake results by flow cytometry. During the incubation, the fluorescence intensity of the cells exposed to ALA or AHA NPs rose from 1 to 6 h, then fell from 6 to 12 h. Moreover, the MFI of AHA NPs was consistently higher than that of free ALA, indicating a better cell uptake capacity. CLSM was then carried out to trace the intracellular distribution of free ALA as well as AHA NPs (Figure S4B). AHA NPs could effectively enter tumor cells, break the hydrazone bond and release the ALA at acidic lysosomes, which was further converted into protoporphyrin (PpIX).

In tumor cells, heme was mainly synthesized in mitochondria by a series of metabolic reactions, with a lot of intermediates and mediators that could modulate endogenous the level of heme.^[^ ^38^, ^51^, ^52^ ^]^ Glycine and succinyl‐CoA were condensed to 5‐amino levulinic acid (ALA) under the catalysis of 5‐aminolevulinic acid synthase (ALAS). ALA was subsequently catalyzed by various porphyrin oxidase enzymes to generate PpIX, which further combined with intracellular Fe^2+^ to generate heme. Artemisinin was triggered by heme to produce ROS, promiscuously alkylated a variety of cellular targets according to prior works from other groups.^[^ ^48^ ^]^ Thus, the internal iron/heme levels were assessed in HepG2 cells. The level of heme as well as intracellular iron was detected in HepG2 cells (Figure 2B,C). The addition of ALA enhanced the level of intracellular heme by influencing the intracellular iron, indicating the amplification of the anticancer efficacy of the ARS together with ALA. Additionally, after co‐incubation with ARS and ARS‐HBZ, the intracellular iron level rose through boosting lysosome‐mediated ferritin degradation and IRP‐mediated translational repression of ferritin, further promoting the subsequent production.^[^ ^26^ ^]^

The intracellular ROS production by AHA NPs was examined using flow cytometry and CLSM with DCFH‐DA as the ROS probe. The results by flow cytometry (Figure S5) indicated that the ARS‐HBZ group produced considerably more ROS than that in ARS group, due to the modification of HBZ to the peroxide bridge bonds, resulting in better reactive activity and ROS production. After the addition of ALA, the ROS production ability of ARS and ARS‐HBZ was further enhanced. Meanwhile, the group of AHA NPs demonstrated the highest fluorescent intensity. After AHA NPs were internalized by the tumor cells, the hydrazone bond broke and released free ALA as well as ARS‐HBZ. ALA generated heme in the mitochondria of tumor cells, which could further catalyze ARS‐HBZ to produce ROS. The data from CLSM also confirmed the production of ROS in Figure 2D. The group of AHA NPs occupied the brightest signal of green fluorescence for DCF and red fluorescence for PpIX.

### 3.3 In vitro anti‐tumor efficacy

**FIGURE 3:**
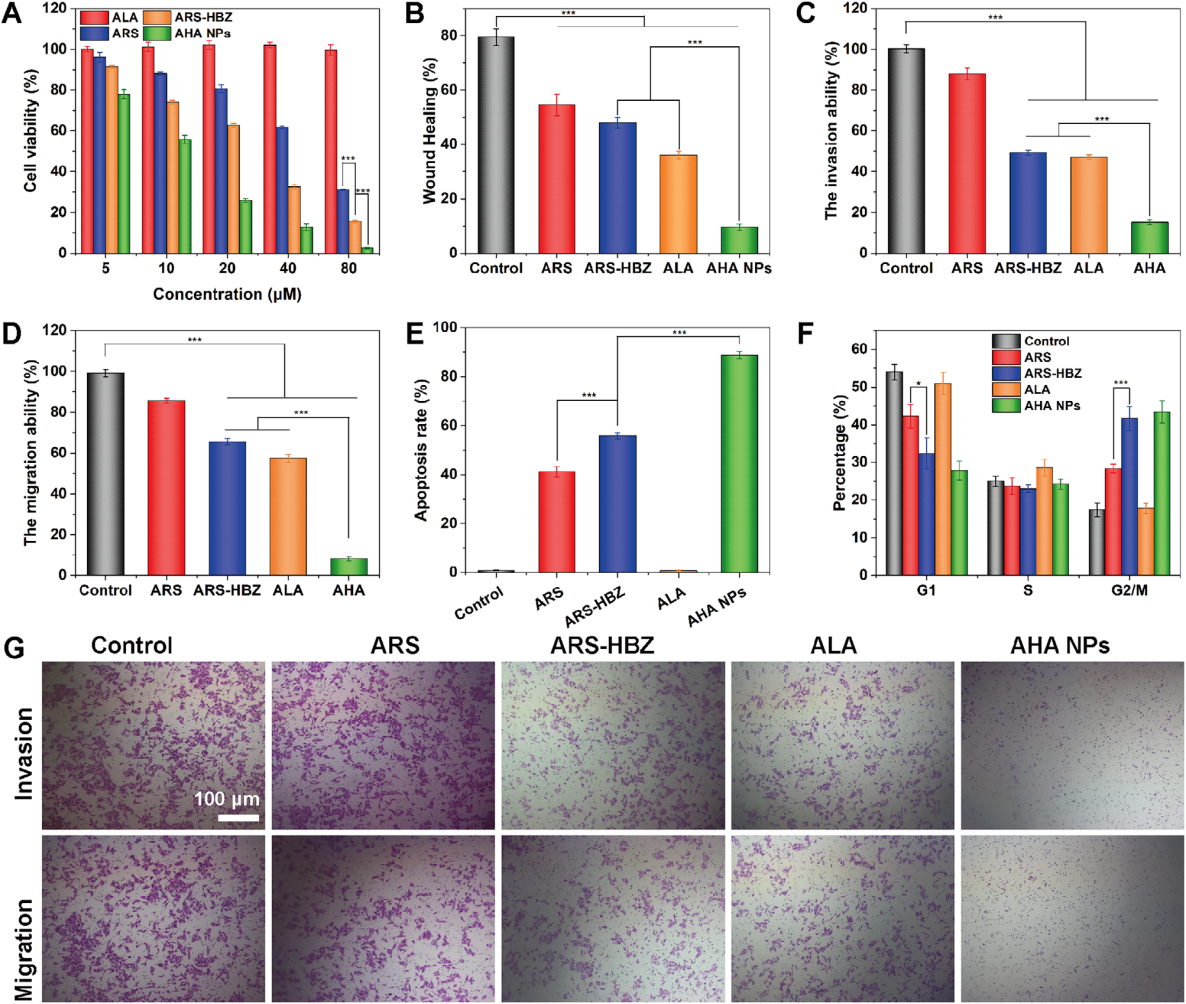
In vitro anti‐tumor mechanism of AHA NPs. (A) The viabilities of HepG2 cells treated with AHA NPs (mean ± SD, *n* = 5). (B) The wound healing ratio of HepG2 cells with different treatments at 24 h. The (C) invasion and (D) migration results of HepG2 cells treated with various formulations. (E) Apoptosis results and (F) cell cycle analysis of HepG2 cells incubated with ARS, ARS‐HBZ, ALA and AHA NPs. (G) Transwell invasion and migration assay of HepG2 cells treated with various formulations. Data are given as mean ± S.D. (*n* = 3). *p < 0.05, **p < 0.01 and ***p < 0.001. The scale bar is 100 µm.

The MTT assay was used to conduct cytotoxicity of ALA, ARS, ARS‐HBZ, and AHA NPs. As shown in Figure 3A, the cell viability in the group of ALA was consistently above 90%, indicating the seldom cytotoxicity. The cytotoxicity increased when treated with higher concentrations of ARS or ARS‐HBZ cell, revealing the dose‐dependent cytotoxicity. Moreover, ARS‐HBZ performed much higher inhibition efficiency than that of ARS. As the peroxide bridge bond of ARS‐HBZ was to produce effective to produce ROS that can alkylate tumor cell proteins, the grafting of HBZ might also enhance the toxicity of artesunate for cancer therapy. The in vitro cytotoxicity of AHA NPs on L02 cells was shown in Figure S6. The cytotoxicity of AHA NPs to L02 cells remained dose‐dependent, but due to the lower Fe^2+^ content in normal cells compared to tumor cells, AHA NPs showed weaker cytotoxicity to normal cells. The group of AHA NPs showed the best cytotoxicity owing to the ROS generation catalyzed by heme.

As previously reported, artemisinin derivatives and iron chelators could help suppress the metastasis of cancer cells.^[^ ^27^, ^53^, ^54^, ^55^ ^]^ Thus, to further examine the impact of AHA NPs in vitro, HepG2 cells were used for wound healing and cell migration tests (Figure S7). As found in Figure 3B, the control group had an apparent healing capacity with 78% of the scratching wounds healed, highlighting the solid metastatic feature of hepatocarcinoma cells. The treatment with ARS and ARS‐HBZ demonstrated a broad inhibiting impact to repair wounds. Due to the strong influence in iron deficiency, ALA might severely impede cell motility. Notably, cells treated with AHA NPs demonstrated limited cell motility with only 8% of wound closure. Transwells were used to further evaluate the impact of AHA NPs on the invading and migration of the HepG2 cells (Figure 3C,D). The majority of the cells in the control group moved to the chamber at the lower surface (Figure 3G). The capacity of the cells to migrate was tolerably inhibited by ARS, ARS‐HBZ, and ALA therapy alone. In particular, the combination treatment with AHA NPs resulted in almost any cells being visible on the lower surface of the transwell chamber. Additionally, ARS‐HBZ showed more efficient than that of ARS in preventing the migration and metastasis of the HepG2 cells, revealing that the migratory and invasion of highly metastatic HepG2 cells could be effectively suppressed by AHA NPs. The above results indicated that the AHA NPs exhibited great potential in suppressing the migration and metastasis of HepG2 cells.

The apoptosis level of HepG2 cells was assessed by flow cytometry using the Annexin V‐FITC/PI labeling method. As shown in Figure 3E, both ARS and ARS‐HBZ groups promoted cell apoptosis, indicating that the modified artesunate was an effective drug for chemotherapy. The apoptosis rate of the ARS‐HBZ group increased to 58.5%, much higher than that of the ARS group (39.2%), further verified that the prepared ARS‐HBZ enhanced its cytotoxicity and promoted cell apoptosis by improving the electronegativity of the peroxide bridge (Figure S8). The apoptosis rate of the AHA NPs group was as high as 88.8%, caused by the ALA released from AHA NPs in HepG2 cells due to the cleavage of the hydrazone bond. The increased level of heme in HepG2 cells promoted the occurrence of chemical kinetic changes in ARS‐HBZ to produce ROS. The generated ROS combined with the cell‐killing ability of ARS‐HBZ to promote HepG2 cell apoptosis.

Flow cytometry was further employed to identify the phase distribution of the cell cycle in HepG2 cells treated with AHA NPs. Figure 3F showed that the control group had 54.0% of cells in the G1 phase and 17.4% in the G2/M phase. The addition of ALA hardly impacted the cell cycle of HepG2 cells. The populations in G1 phase in the groups of ARS, ARS‐HBZ, and AHA NPs were at 43.2%, 32.4% and 27.8%, respectively, whereas the populations in G2/M phase were more significant at 28.4%, 41.7% and 43.4%. After incubation with ARS, the number of cells in G1 phase dropped while the number of G2/M phase cells raised, illustrating that ARS greatly influenced the prophase and mitotic phases of the cell cycle (Figure S9). These findings aligned with other literature previously that demonstrated the ability of artemisinin derivates to stop the cell cycle in the G2/M phase.^[^ ^56^, ^57^ ^]^ From the data, the prepared ARS‐HBZ had higher block ability in cell cycle than that of ARS, indicating a promising and excellent vista of artemisinin derivative for subsequent clinical research.

### 3.4 In vivo anti‐tumor efficacy

**FIGURE 4:**
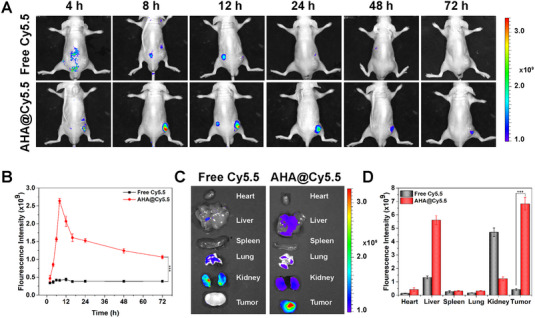
Biodistribution of AHA NPs in vivo. (A) In vivo fluorescence imaging of free Cy5.5 and AHA@Cy5.5 in HepG2 tumor‐bearing mice. (B) Semiquantitative fluorescence intensity statistics of tumor tissues at different time. (C) Ex vivo fluorescence images of major organs and tumors at 24 h post‐injection. (D) Quantification of the fluorescent signals associated with the major organs and tumors. Data is given as mean ± SD (*n* = 3). *p < 0.05, **p < 0.01 and ***p < 0.001.

Real‐time NIR fluorescence imaging was used to monitor the biodistribution of AHA NPs for in vivo at various time points in mice bearing with HepG2 tumors (Figure 4A,B). After intravenous administration of free Cy5.5 and AHA@Cy5.5, free Cy5.5 was primarily dispersed in kidney tissue with slight accumulation to tumor, and the intensity of the fluorescence at the tumor location barely changed after 72 h, demonstrating low tumor‐targeting selectivity. Because the metabolic pathway of Cyanine was mainly through the kidney, the fluorescence of free Cy5.5 was immediately distributed throughout the body after intravenous injection, and then gradually cleared through renal excretion.^[^ ^58^, ^59^ ^]^ AHA NPs usually accumulated in liver tissue in addition to excellent tumor targeting ability. The fluorescence signal in tumor areas of mice treated with AHA@Cy5.5 reach the most after 8 h and persisted in the tumor tissue for a substantial amount of time within 72 h at least, and the central accumulation became apparent in the tumor. Major organs from each mouse were collected 24 h after injection. Figure 4C,D provided the quantification of essential tissues along with fluorescence pictures. The accumulation and excretion of free Cy5.5 majorly occurred in the kidney, with just minor tumor aggregation. The AHA@Cy5.5 group, on the other hand, had fluorescence intensities in their tumors that were 15.9 times higher than those of free Cy5.5 groups. All of these findings showed that AHA NPs had a long blood circulation and could target the tumor regions, reducing the systemic toxicity of chemotherapeutic agents.

**FIGURE 5:**
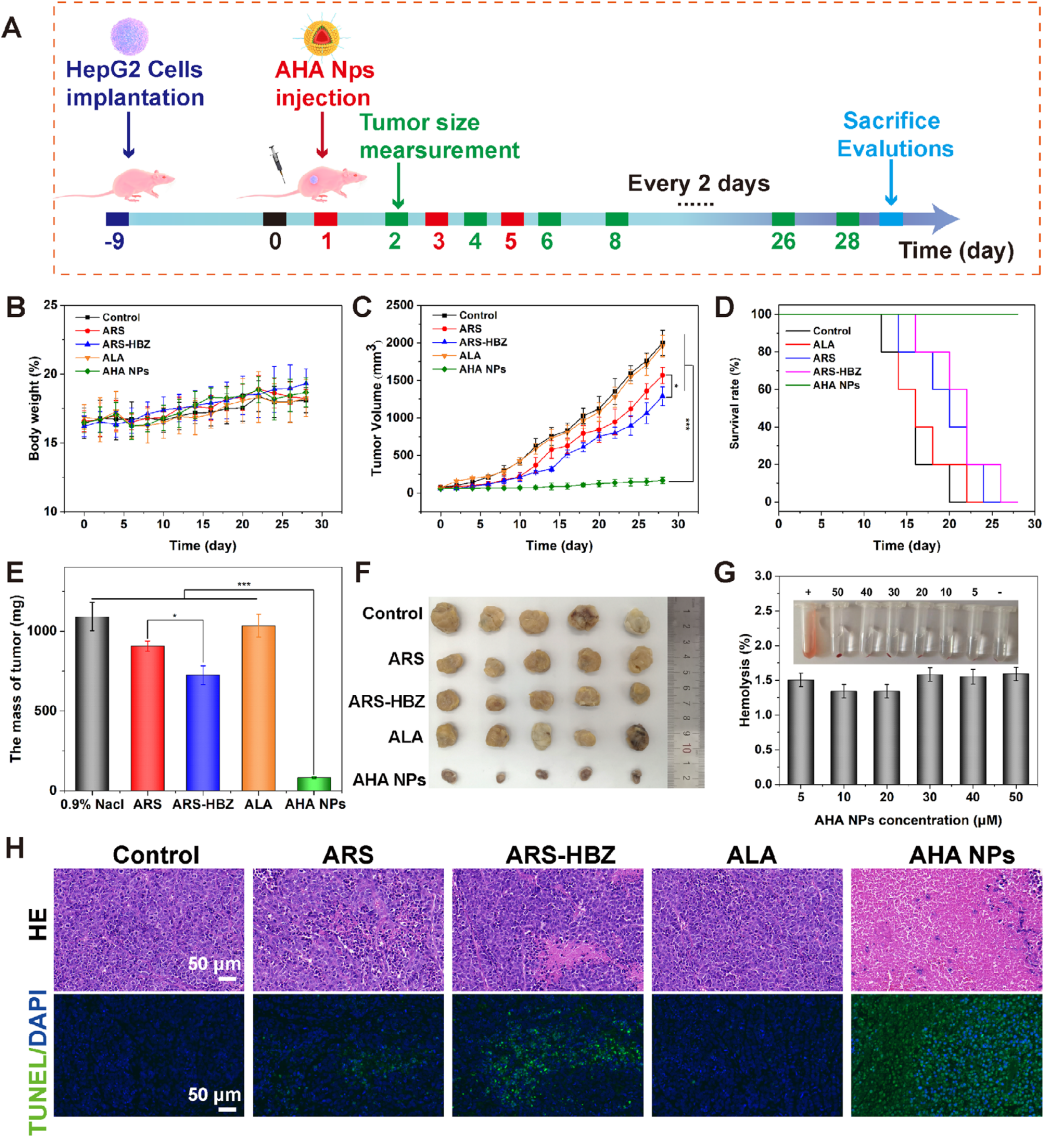
Anti‐tumor efficiency of AHA NPs in vivo. (A) Schematic illustration of tumor model establishment and therapy process of AHA NPs. Variations in (B) body weight and (C) tumor volume of the mice treated with 0.9% NaCl, ALA, ARS, ARS‐HBZs and AHA NPs. (D) The survival rates of the mice after different treatments. (E) The weight and (F) morphology of the harvested tumors on 28th day. (G) Hemolytic activity of AHA NPs in different concentrations after 2 h of incubation with erythrocytes. (H) The images of tumor tissue sections stained by H&E and TUNEL. Data are given as mean ± S.D. (*n* = 5). *p < 0.05, **p < 0.01 and ***p < 0.001. Scale bar = 50 µm.

The anticancer efficiency of AHA NPs was further studied in vivo on HepG2 tumor‐bearing mice based on the in vitro test findings. As depicted in Figure 5A, HepG2 cells were implanted into BALA/c‐nu mice, AHA NPs were intravenously injected three times, and the weight and tumor volume were measured every two days. All the mice were sacrificed for further analysis at the 28th day. Figure 5C showed that the control group suffered from rapid tumor development throughout anticancer therapy process. The ALA group showed almost no treatment effect on tumors. The group treated with ARS and ARS‐HBZ resulted in a limited amount of inhibition of the growth of the tumor volume, suggesting that ARS and ARS‐HBZ chemotherapy alone was less effective to suppress the growth of the tumor. The tumor inhibitory of ARS‐HBZ was better than that of ARS, consistent with previous experimental evidence that ARS‐HBZ had better reactivity of the peroxide bridge bond. Due to their anticancer impact and improved tumor enrichment, AHA NPs performed the best antitumor capacity among other groups. The tumors harvested on the 28th day were weighed and photographed (Figure 5E,F) The outcomes were consistent with the data on tumor development in Figure 5C. As a nanosized artemisinin product, AHA NPs might effectively accumulate in tumors because of the EPR effect and longer blood circulation times. After internalization, ALA and ARS‐HBZ could be released rapidly under the cleavage of hydrazone bonds induced by acidic surroundings in lysosomes. Additionally, ALA increased the intracellular level of heme through a series of enzymatic catalytic reactions in mitochondria, facilitating the generation of ROS by combining with ARS‐HBZ. The release of ROS and ARS‐HBZ would subsequently cause membrane damage and cell apoptosis to achieve extraordinary antitumor efficacy. Additionally, the body weight of each mouse kept stable during the treatment procedures, suggesting that the AHA NPs formulations were safe and promising for clinical applications (Figure 5B). The AHA NPs also performed noteworthy hemocompatibility and low level of hemolysis (2% hemolysis) at 50 µm (Figure 5G). Additionally, the mice in the AHA NPs groups lived for more than 28 days before they were sacrificed. In contrast, all the mice in the other groups were perished or exceeded the predetermined end‐value of 1500 mm^3^ in 16–28 days after treatment (Figure 5D). In brief, the ROS level in the group treated with AHA NPs was amplified by ARS‐HBZ via interactions with heme, could effectively extend the mice's survival time.

H&E and TUNEL staining were carried out to evaluate the proliferation and apoptosis of HepG2 cells. As shown in Figure 5H, the tumor tissue of control and the ALA group exhibited malignant hyperplasia and were tightly packed. In contrast, several tumor cells in the group treated with free ARS atrophied as a result of nuclear pyknosis and chromatin condensation. The ARS‐HBZ group gave more noticeable necrosis and cell shrinkage. The sample treated with AHA NPs showed the highest amount of apoptotic tumor cells, together with pyknosis and ruptured cell membranes, demonstrating exceptional in vivo anticancer activity. TUNEL labeling was also used to examine tumor apoptosis following the 28‐day therapy. In accordance with the H&E staining figures, the control and ALA groups did not exhibit any obvious apoptotic markers. However, a small amount of apoptosis was found in the ARS and ARS‐HBZ groups. The TUNEL positive cells were most prevalent in the AHA NPs group (Figure 5H).

### 3.5 Biosafety

**FIGURE 6:**
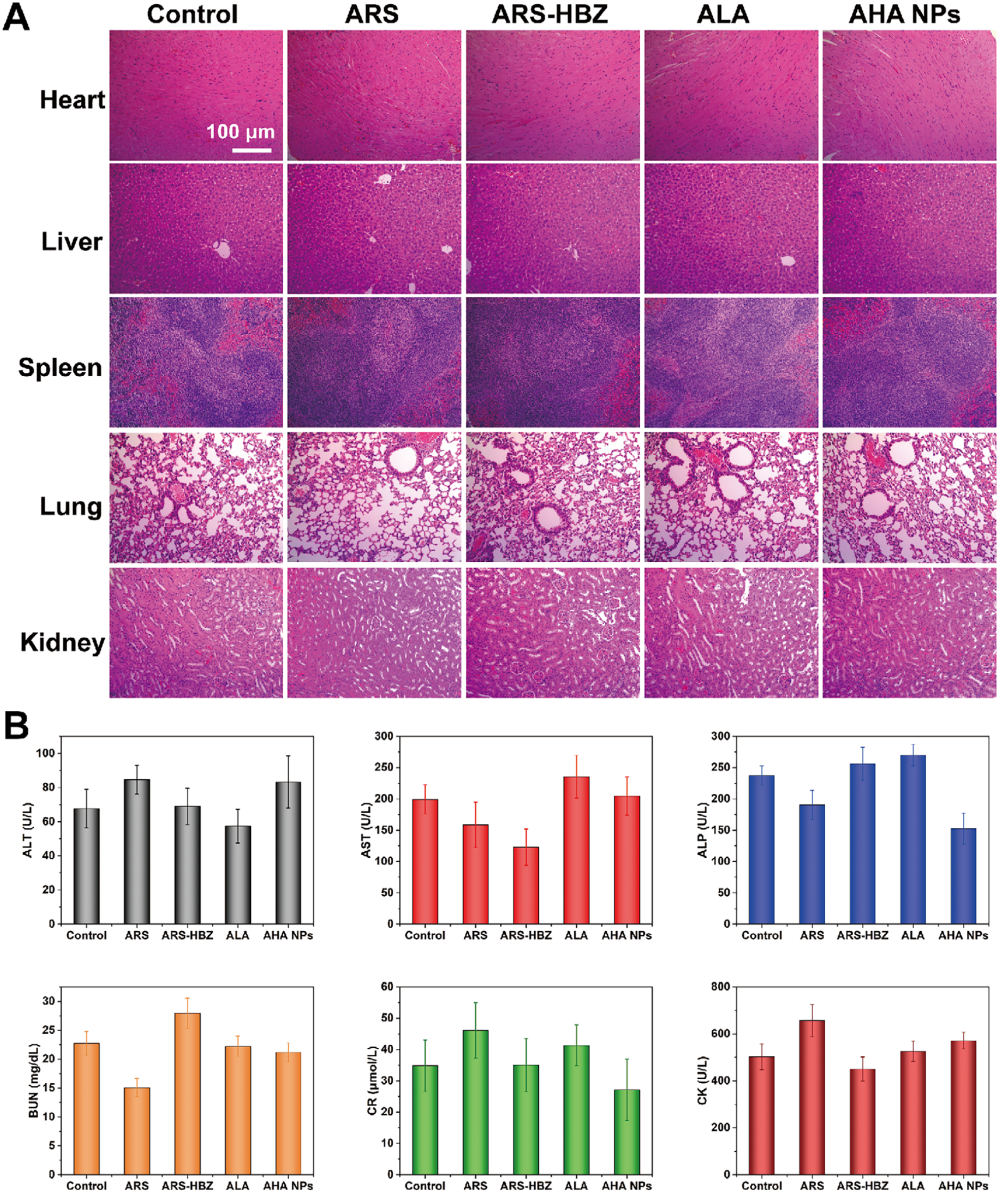
Biosafety of AHA NPs in vivo. (A) Hematoxylin and eosin (H&E) staining results of normal organs (heart, liver, spleen, lung and kidney) from mice on the 28th day. (B) Serum biochemical analysis of the mice. Data are given as mean ± S.D. (*n* = 5). *p < 0.05, **p < 0.01 and ***p < 0.001. Scale bar = 100 µm.

To determine the biosafety of AHA NPs for clinical applications, the H&E staining of organ slices and serum biochemical analyses were carried out. The tumor‐bearing mice were sacrificed. The heart, liver, spleen, lung, and kidney were removed and stained with H&E. The findings demonstrated that none of the therapies, including those using ALA, ARS, ARS‐HBZ, and AHA NPs, appeared to have any adverse effects on these significant organs (Figure 6A). Additionally, biochemical tests were performed to detect the hepatotoxicity (the concentration of ALT, AST or ALP), nephrotoxicity (BUN or CRE), and cardiac toxicity (CK). The hepatic, renal, and myocardial function indices in each treatment group were similar with the control group (Figure 6B), demonstrating the safety potential of AHA NPs as an anticancer treatment.

## 4 CONCLUSIONS

In this study, we reported a novel pH‐responsive and ROS‐self‐supplied AHA NPs for excellent chemotherapy and CDT. The nanosized artemisinin derivatives outperformed the free medication regarding anticancer activity in the animal model of liver cancer. The AHA NPs showed a long blood circulation and tumor targeting ability via the EPR effect. After internalization by tumor cells, ALA and ARS‐HBZ could rapidly release after the cleavage of hydrazone bonds in acid surroundings to further inhibit the cell proliferation. Compared to artesunate, ARS‐HBZ showed better ROS production capacity under the electron absorption effect of HBZ. Additionally, ALA increased the intracellular level of heme through a series of enzymatic catalytic reactions in mitochondria, which promoted the generation of ROS to induce cell apoptosis. The AHA NPs would generate more efficient medications based on artemisinin derivatives for treating liver cancer because of their sensitive drug release features and synergistic chemotherapy. The chemotherapy effect and biological half‐life of artemisinin were greatly improved by facilitation of peroxide bridge using the nanoprecipitation method. Our study shed light on the acceleration of artemisinin prodrugs in clinical transformation prospects.

## AUTHOR CONTRIBUTIONS

Junjie Tang: Conceptualization, Methodology, Formal analysis, Data curation; Yadong Liu: Investigation, Formal analysis; Yifang Xue: Investigation, Methodology. Zhaozhong Jiang: Investigation. Baizhu Chen: Investigation, Writing, Review and editing, Supervision; Jie Liu: Conceptualization, Resources, Project administration, Supervision.

## CONFLICT OF INTEREST STATEMENT

The authors declare no conflicts of interest.

## ETHICAL APPROVAL STATEMENT

All the animal procedures were performed in compliance with the Regulations for the Administration of Affairs Concerning Experimental Animals of China, and all animal experiments were approved by the Animal Care and Use Committee of Sun Yat‐sen University (Permit No. SYSU‐IACUC‐2022‐000796).

## Supporting information

Supporting Information

## Data Availability

All data of this work are present in the article and Supporting Information. The other data that support the findings of this work are available from the corresponding author upon reasonable request.
