# Supplementary material for: Endoperoxide‐enhanced self‐assembled ROS producer as intracellular prodrugs for tumor chemotherapy and chemodynamic therapy
Source: Exploration (Beijing). 2024 Feb 9;4(4):20230127. doi: 10.1002/EXP.20230127 (PMC11335464; doi:10.1002/EXP.20230127)
Supplement: Supplementary file 1 — Supporting Information [file EXP2-4-20230127-s001.docx]

**Endoperoxide-enhanced Self-Assembled ROS Producer as Intracellular Prodrugs for Tumor Chemotherapy and Chemodynamic Therapy**

**JunJie Tang^1^, Yadong Liu^1^, Yifan Xue^1^, Zhaozhong Jiang^3^, Baizhu Chen^1,2,*^, Jie Liu^1,*^**

^1^ *School of Biomedical Engineering, Shenzhen Campus of Sun Yat-Sen University, No. 66, Gongchang Road, Guangming District, Shenzhen, Guangdong 518107, People’s Republic of China;*

*^2^ Guangdong Provincial Key Laboratory of Sensor Technology and Biomedical Instrument, Sun Yat-Sen University, Guangzhou, China*

*^3^ Department of Biomedical Engineering, Integrated Science and Technology Center, Yale University, 600 West Campus Drive, West Haven, Connecticut 06516, United States*

*Corresponding author.

Email: [chenbzh8@mail.sysu.edu.cn](mailto:chenbzh8@mail.sysu.edu.cn); liujie56@mail.sysu.edu.cn

**Methods**

***In vitro* stability of** **AHA NPs:** AHA NPs were dispersed in PBS (pH 7.4, 10 mM) containing 10% (v/v) of fetal bovine serum (FBS), and incubated at 37 ℃ with continuous shaking at 100 rpm for 96 h. Samples were withdrawn from the solutions at pre-determined time intervals for DLS analysis.

**Cellular uptake:** HepG2 cells were seeded in 6-well plates and confocal bottom plates at a density of 3 × 10^5^ per well. For an overnight incubation, the cells were treated with fresh medium containing free ALA and AHA NPs (ALA concentration = 40 μM) for 1-12 h. And then the cells were washed with PBS for three times. The fluorescence intensity of PpIX in single cell suspension was measured by flow cytometry and CLSM.

**Cellular ROS generation:** To investigate intracellular ROS production, HepG2 cells (1×10^5^ cells/mL) were plated at 6-well culture plates for overnight incubation. Thereafter, the adherent cells were randomly divided into 7 groups as control, ALA, ARS, ARS-HBZ, ARS+ALA, ARS-HBZ+ALA and AHA NPs groups. After the HepG2 cell groups were incubated under their respective treatment conditions for 6 h, the culture medium was replaced with 20 µM DCFH-DA to react with intracellular ROS for 20 min. Subsequently, the DCF fluorescence intensity of all groups in single cell suspension was quantitatively measured by flow cytometry.

**The survival rate of tumor-bearing mice:** To study antitumor activity in vivo, the tumor-bearing mice with tumor volume reaching approximately 100 mm^3^ were randomly divided into six groups (n = 5): (i) 0.9% NaCl (control), (ii) free ARS, (iii) free ARS-HBZ, (iv)free ALA, (v) AHA NPs. Different drug formulations with 20 mg·kg^-1^ ARS and 8 mg·kg^-1^ ALA were injected intravenously to the mice on Day 0, 3, and 6, respectively. The tumor volume changes of the mice were recorded every two days throughout the treatment. The Kaplan-Meier survival curves were obtained by the percent mice when the tumor volume >1500 mm^3^ or the mice died.

**Hemolysis assay:** The blood compatibility of AHA NPs was assessed by hemolysis assay. Briefly, erythrocytes (2×10^8^ RBCs) were incubated with blank AHA NPs (5-50 µM) for 2 h. After centrifugation at 1000 rpm for 10 min, the absorbance of suspensions at 410 nm was measured by a microplate reader. Triton-100 (1%, w/v) and PBS were used as the positive and negative control, respectively. Hemolysis value was calculated by the equation below:

$$\mathrm{Hemolysis}\left( \% \right)=\frac{A_{sample}-A_{PBS}}{A_{Triton}-A_{PBS}}\times100\%$$

where $A_{sample}$, $A_{PBS}$ and$A_{Triton}$ represent the absorbance intensity values of the supernatants from RBCs treated with the NP samples, PBS and Triton X-100, respectively.


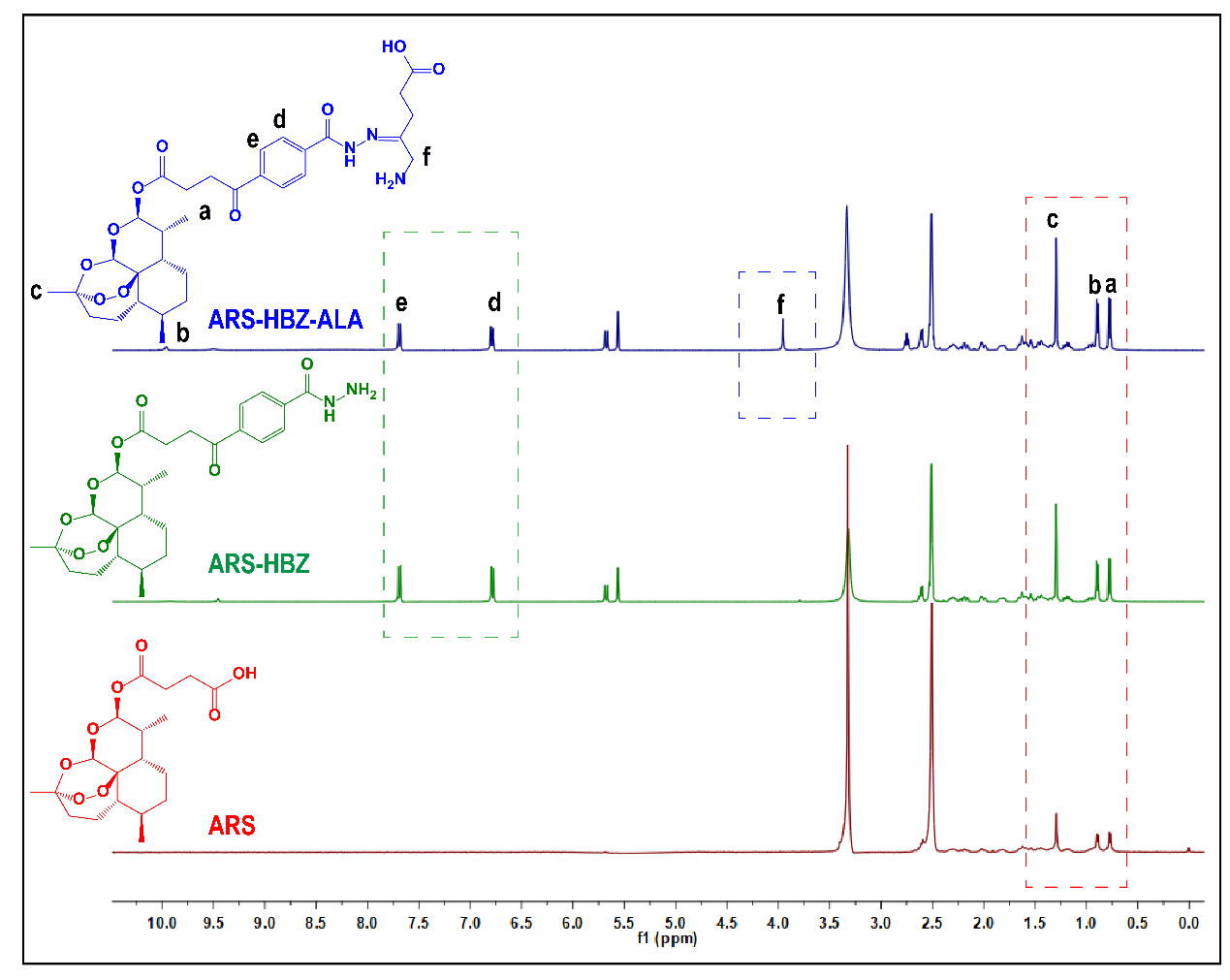


**Figure S1.** ^1^H NMR spectra of ARS, ARS-HBZ and ARS-HBZ-ALA.





**Figure S2.** Average particle size vs time for AHA NPs incubated in PBS (10 mM, pH=7.4) containing 10% of FBS.





**Figure S3.** ROS generation by Fe^2+^ and heme activation of AHA NPs.


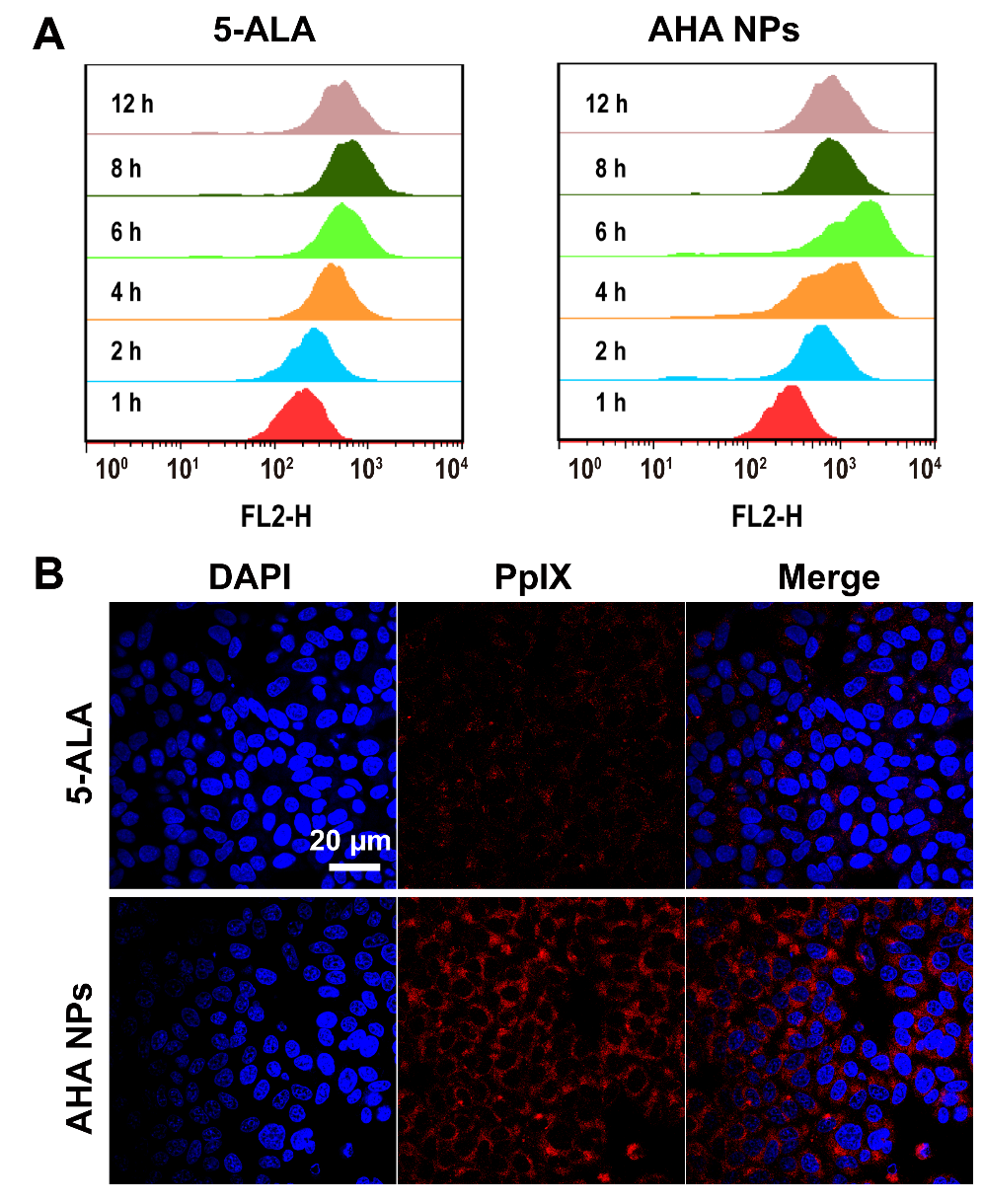


**Figure S4.** (A) Fluorescence histograms of HepG2 cells treated with free 5-ALA and AHA NPs, (B) CLSM images of the intracellular distribution of free ALA and AHA NPs after incubation with HepG2 cells for 6 h.


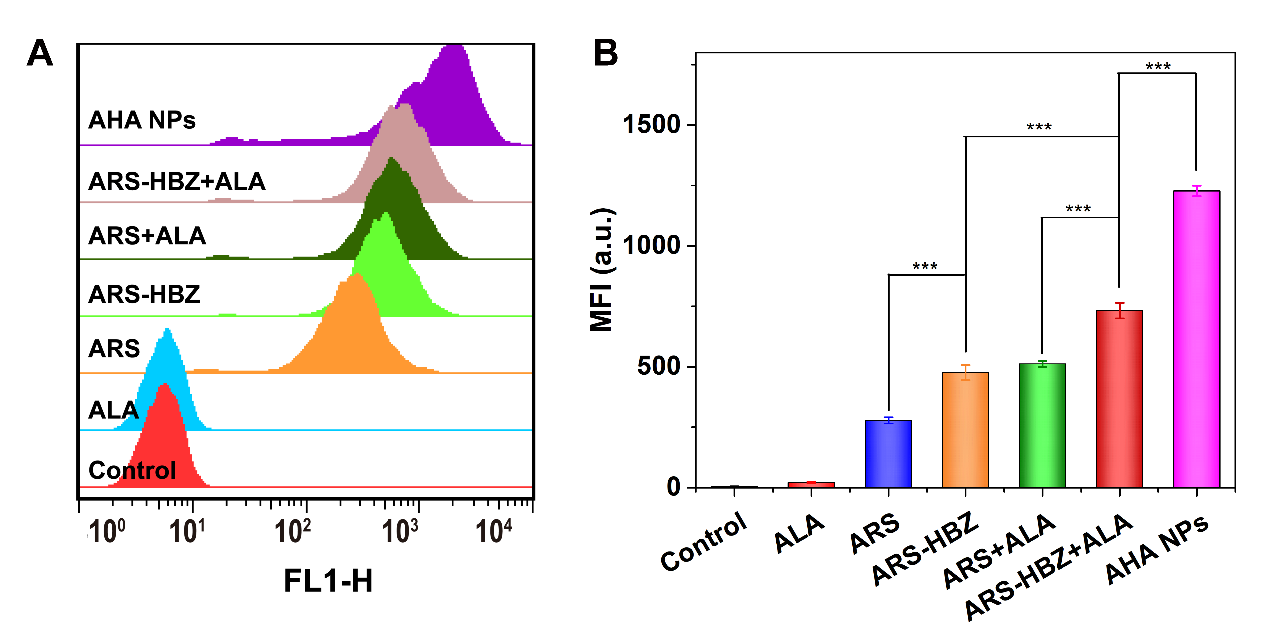


**Figure S5**. (A) Fluorescence histograms of the ROS produced by AHA NPs. (B) The intracellular ROS MFI values of HepG2 cells treated with AHA NPs. **p < 0.05, **p < 0.01 and ***p < 0.001*.





**Figure S6.** The viabilities of L02 cells treated with AHA NPs (mean ± SD, n=5).


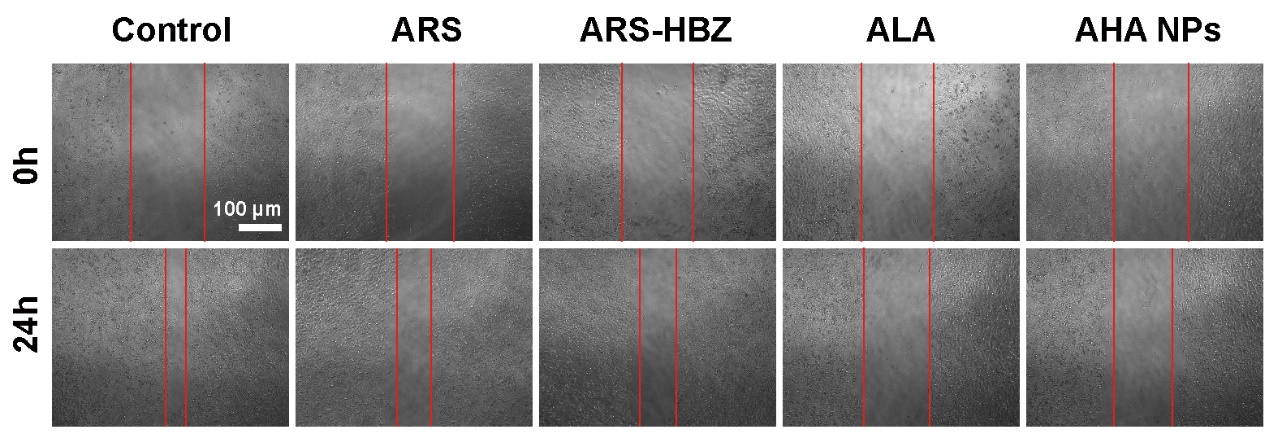


**Figure S7**. The images of wound HepG2 cells treated with various formulations. Scale bar = 100 µm.


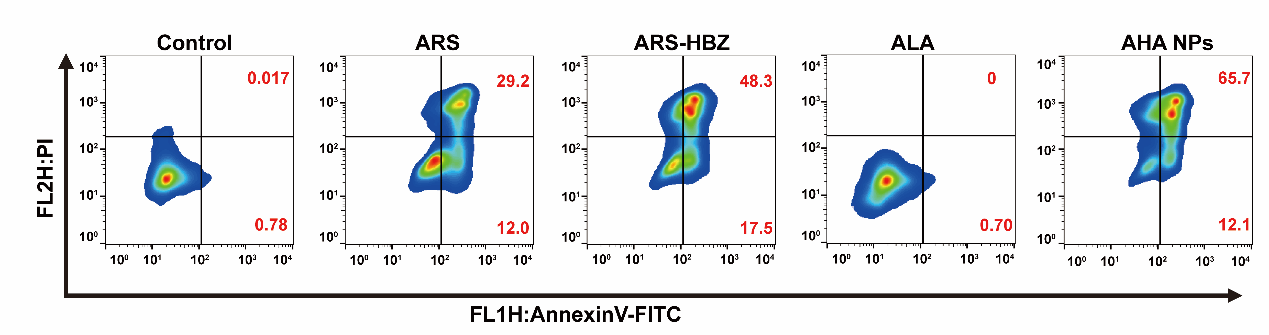


**Figure S8.** Apoptosis results of HepG2 cells incubated with ARS, ARS-HBZ, ALA and AHA NPs. **p < 0.05, **p < 0.01 and ***p < 0.001*.


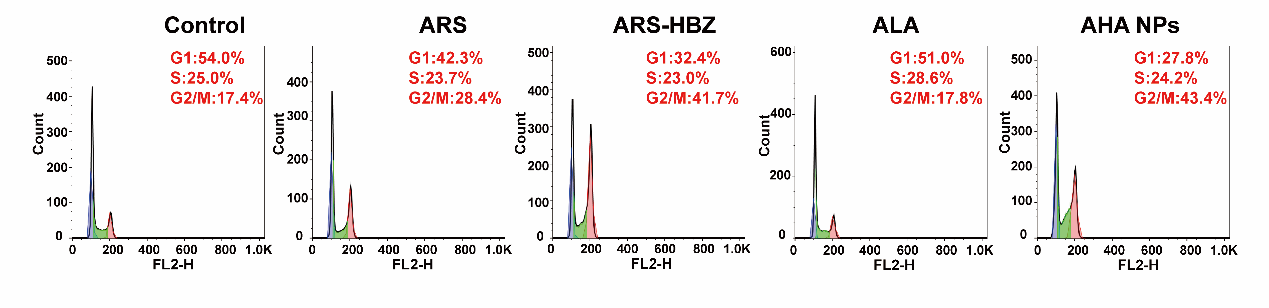


**Figure S9.** Cell cycle results of HepG2 cells incubated with ARS, ARS-HBZ, ALA and AHA NPs. **p < 0.05, **p < 0.01 and ***p < 0.001*.
